# Mechanism of *KIT* gene regulation by GATA1 lacking the N-terminal domain in Down syndrome–related myeloid disorders

**DOI:** 10.1038/s41598-022-25046-z

**Published:** 2022-11-29

**Authors:** Rika Kanezaki, Tsutomu Toki, Kiminori Terui, Tomohiko Sato, Akie Kobayashi, Ko Kudo, Takuya Kamio, Shinya Sasaki, Koji Kawaguchi, Kenichiro Watanabe, Etsuro Ito

**Affiliations:** 1 Department of Pediatrics, Hirosaki University Graduate School of Medicine, 5 Zaifucho, Hirosaki, Aomori 036-8562 Japan; 2 Department of Hematology and Oncology, Shizuoka Children’s Hospital, Shizuoka, Japan; 3 Department of Community Medicine, Hirosaki University Graduate School of Medicine, Hirosaki, Japan

**Keywords:** Acute myeloid leukaemia, Transcription

## Abstract

Children with Down syndrome (DS) are at high risk of transient abnormal myelopoiesis (TAM) and myeloid leukemia of DS (ML-DS). *GATA1* mutations are detected in almost all TAM and ML-DS samples, with exclusive expression of short GATA1 protein (GATA1s) lacking the N-terminal domain (NTD). However, it remains to be clarified how GATA1s is involved with both disorders. Here, we established the K562 GATA1s (K562-G1s) clones expressing only GATA1s by CRISPR/Cas9 genome editing. The K562-G1s clones expressed *KIT* at significantly higher levels compared to the wild type of K562 (K562-WT). Chromatin immunoprecipitation studies identified the GATA1-bound regulatory sites upstream of *KIT* in K562-WT, K562-G1s clones and two ML-DS cell lines; KPAM1 and CMK11-5. Sonication-based chromosome conformation capture (3C) assay demonstrated that in K562-WT, the − 87 kb enhancer region of *KIT* was proximal to the − 115 kb, − 109 kb and + 1 kb region, while in a K562-G1s clone, CMK11-5 and primary TAM cells, the − 87 kb region was more proximal to the *KIT* transcriptional start site. These results suggest that the NTD of GATA1 is essential for proper genomic conformation and regulation of *KIT* gene expression, and that perturbation of this function might be involved in the pathogenesis of TAM and ML-DS.

## Introduction

Transient abnormal myelopoiesis (TAM) is a disease associated with an increase in abnormal blast cells expressing erythrocyte and megakaryocyte-specific genes^1^. TAM occurs in about 10% of newborns with Down syndrome (DS)^2^. Given that the liver is a hematopoietic organ during the fetal period and TAM blast cells decrease after birth, the fetal liver may produce signals that support TAM blast cell proliferation. While most TAM cases result in spontaneous remission, about 20% of TAM patients develop myelodysplastic syndrome (MDS) and acute megakaryoblastic leukemia (DS-AMKL) within 4 years after birth, referred to as myeloid leukemia of DS (ML-DS)^2,3^.

GATA1 is a transcription factor expressed in erythrocytes, megakaryocytes, eosinophils and mast cells. GATA1 has an N-terminal transcription activation domain (NTD) and two Zn finger domains, and binds to the DNA motif; (A/T)GATA(A/G) to regulate transcription of many target genes^4^. In erythrocyte and megakaryocyte lineages, GATA1 is an essential factor for differentiation^5,6^. *GATA1* mutations are detected in almost all TAM and ML-DS cases^7–11^. These mutations lead to exclusive expression of short GATA1 (GATA1s) protein lacking the NTD. In addition, a few cases with Diamond-Blackfan anemia (DBA)-like phenotype have germline mutations in *GATA1*, which also lead to exclusive expression of GATA1s^12–14^. Introduction of GATA1s leads to abnormal proliferation of megakaryocytic cells in mouse model experiments^15,16^. In experiments using human iPS cells, *GATA1*-truncation mutations exhibit impaired erythroid potential, enhanced megakaryopoiesis and myelopoiesis^17^ or aberrant differentiation of megakaryocytes^18,19^. Whole-exome and targeted sequencing analysis of TAM indicates that *GATA1* mutation is sufficient to cause TAM in DS neonates^20,21^. However, it remains to be clarified how GATA1s is involved with TAM and ML-DS blast cell proliferation.

The proto-oncogene *KIT* encodes a transmembrane type III tyrosine kinase, which is a receptor for stem cell factor (SCF). Fetal hepatocytes that support expansion of hematopoietic stem cells express cytokines including SCF^22–24^. *KIT* and several genes, which are repressed after GATA1 induction in the murine system, are highly expressed in ML-DS compared with Non-DS AMKL^25^. Given that *GATA1* mutations are found in only about 10% of non-DS AMKL^26^, these results suggest that GATA1s fails to properly regulate GATA1 target genes including *KIT.* SCF/KIT signaling has an essential role in the proliferation and survival of blast cells in the DS-related myeloid disorders^27^. In addition, pharmacological KIT inhibition targeted preleukemic stem cells with trisomy 21 and *GATA1* mutation, and inhibits leukemic progression^28^. Nevertheless, it has not been shown how GATA1s is involved in the regulation of *KIT* expression.

Previously, we have introduced wild and various GATA1 mutants into the ML-DS cell line KPAM1^27^ and found that the cells were severely growth suppressed by GATA1^29^. However, this made it difficult to obtain sufficient numbers of cells for subsequent experiments. Therefore, we first performed CRISPR/Cas9 genome editing on K562 to established K562 GATA1s (K562-G1s) clones that express only GATA1s, in order to determine what target gene expression changes are caused by loss of the NTD. The K562-G1s clones expressed *KIT* at higher levels compared to the wild type K562 (K562-WT). We found that both GATA1 and GATA1s were bound upstream of *KIT* by chromatin immunoprecipitation (ChIP) studies. Sonication-based chromosome conformation capture (3C) assay revealed a difference in the conformation of *KIT* between the K562-WT and a K562-G1s clone. Furthermore, a ML-DS cell line and primary TAM cells had the similar genomic conformation of *KIT* to the K562-G1s clone. These results suggest that the lack of the NTD in GATA1 alters the genomic structure of *KIT* and dysregulates *KIT* expression, leading to abnormal proliferation of TAM blast cells.

## Results

### Characterization of the K562-G1s clones

**Figure 1:**
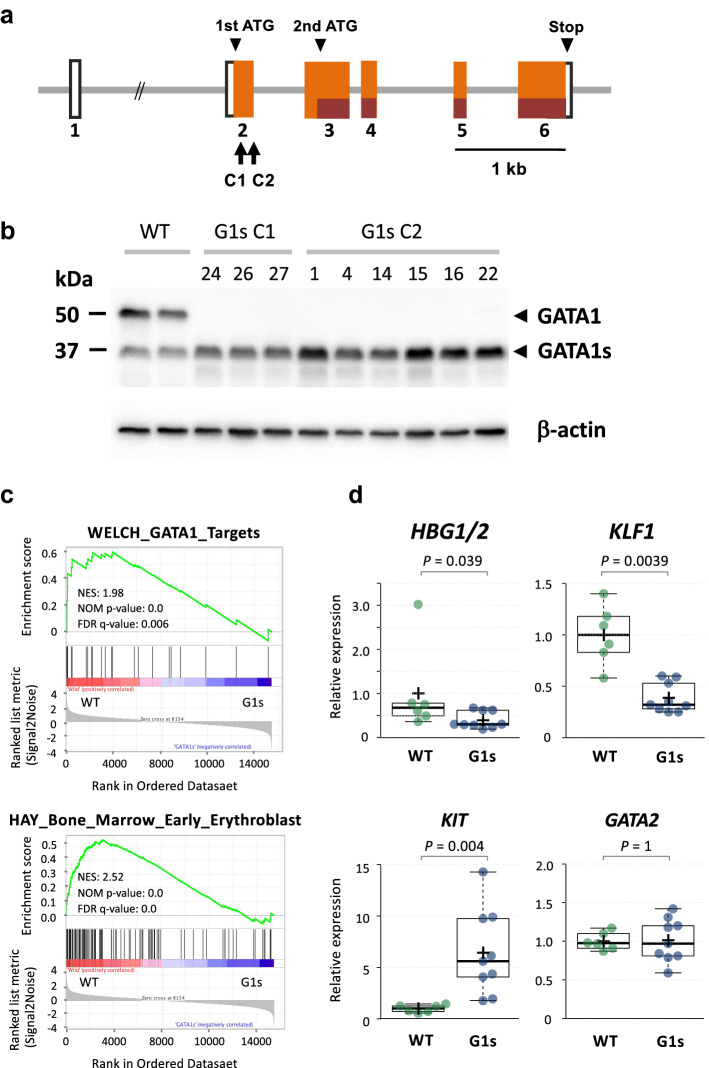
Overview of genome editing of *GATA1* gene by CRISPR/Cas9 system. (**a**) Gene structure diagram of G*ATA1*. The arrows on C1 and C2 indicate the target positions of the two guide RNAs. (**b**) Detection of GATA1 protein in K562-G1s clones obtained by *GATA1* genome editing. Western blotting was performed with anti-GATA1 antibody (D24E4, Cell signaling technology), which could detect both full length GATA1 (45 kDa) and GATA1s (37 kDa) and monoclonal antibody against β-actin (Sigma-Aldrich). Images were obtained using ChemiDoc MP (Bio-Rad Laboratories). Mutations in all clones caused loss of full-length GATA1 production but allowed expression of GATA1s protein from the second translation initiation codon. (**c**) GSEA for the gene sets with differential expression between K562-WT cells and K562-G1s clones. NES, normalized enrichment score; NOM, nominal; FDR, false discovery rate. (**d**) Expression levels of four representative GATA1 target genes in six K562-WT single clones and nine K562-G1s clones by qRT-PCR. Data points were added with dots, and sample means were indicated by crosses. The expression levels of each gene are shown relative to the mean values of the K562-WT single clones, which is set at 1. The *P*-values of the Mann–Whitney U test were noted.

To compare the function of GATA1s with GATA1, we introduced *GATA1* mutations into K562 cells by genome editing, which caused exclusive expression of GATA1s (Fig. 1a–b, Supplemental Fig. S1 and Table S1). Naumann et al.^30^ have reported that K562 has two X chromosomes, one of which is del(X)(p11). *GATA1* is located at chrX p11.23 within this deleted region, and in fact, in each of the K562-G1s clones, only one type of signal was detected for the *GATA1* sequence (Supplemental Fig. S1). To investigate the differences in expression profiles, we performed RNA-seq of nine K562-G1s clones and K562-WT cells. Gene set enrichment analysis (GSEA)^31^ revealed that GATA1 target genes as well as early erythroblast feature genes were down-regulated in the K562-G1s clones (Fig. 1c and Supplemental Fig. S2). Consistent with the RNA-seq results above, qRT-PCR of representative GATA1 target genes showed that *HBG1/2* and *KLF1* were down-regulated in K562-G1s clones, while *KIT* expression was 6.4-fold higher in K562-G1s clones (*P* = 0.004) (Fig. 1d). In contrast, there was no significant difference in the expression level of GATA2 between K562-G1s clones and K562-WT. The decreased expression of *HBG1/2* and *KLF1* can be understood as an effect of the loss of the transcriptional activation domain of GATA1. However, it is interesting to note that GATA1s that has lost its transcriptional activation domain by genome editing induced *KIT* expression, even though the expression of *GATA2*, which is thought to be important for *KIT* expression^32^, was not increased. In addition, *KIT* is abundantly expressed in TAM and ML-DS cells and SCF/KIT signaling has an essential role in the proliferation and survival of blast cells^27^. Therefore, we focused on the mechanism of regulation of *KIT* expression by GATA1 and GATA1s.

### GATA1s is not involved in suppression of *KIT* expression

**Figure 2:**
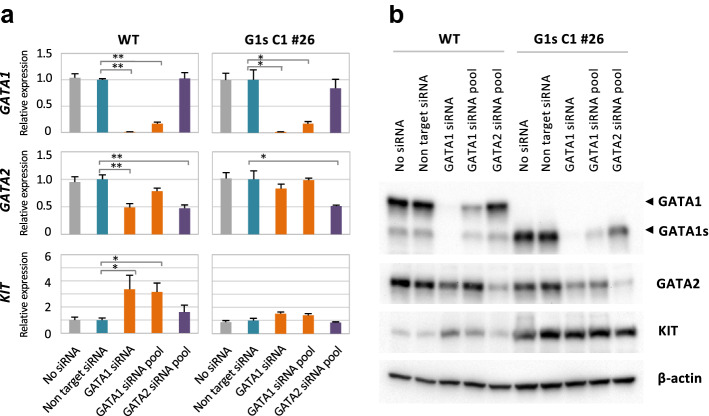
Changes in *KIT* gene expression by *GATA1* siRNAs. (**a**) qRT-PCR analysis was performed for K562-WT (left) and one of the K562-G1s clone, C1 #26 (right). The vertical axis represents a relative value with each value of non-target siRNA set at 1. Results are averages of three independent experiments. Error bars represent standard deviation. *P < 0.05, **P < 0.01 (two-side t-test). (**b**) Representative immunoblots of siRNA experiments with K562-WT (left) and G1s C1 #26 (right). Western blotting was performed with anti-GATA1 antibody (D24E4, Cell signaling technology), anti-GATA2 (AF2046, R&D) anti-c-Kit (SC-17806, Santa Cruz) and anti-β-actin antibody (Sigma-Aldrich). Images were obtained using ChemiDoc MP.

We used *GATA1* knockdown experiments with small interfering RNA (siRNA) to determine whether GATA1 or GATA1s were directly related to *KIT* expression regulation. *GATA1* siRNAs worked effectively to suppress *GATA1* expression in both K562-WT and K562-G1s C1 #26, a K562-G1s clone (Fig. 2a upper panel and b). *GATA2* is a gene whose expression is suppressed by GATA1^33^, but no increase in *GATA2* expression was observed by *GATA1* siRNAs in this experiment (Fig. 2a middle panel and b). *GATA1* knockdown in K562-WT showed an approximate threefold increase in *KIT* expression at the mRNA level (*P* < 0.05) (Fig. 2a left lower panel), although *GATA2* knockdown did not show any significant changes. In contrast, in K562-G1s C1 #26, KIT expression remained high even after *GATA1* knockdown, and no changes in the expression level was observed at both the mRNA and protein levels (Fig. 2a right lower panel and b). These results indicate that KIT expression in K562 may be repressed by GATA1, but that its function may be lost due to the absence of NTD.

### GATA1 and GATA1s bind upstream of the *KIT* gene

**Figure 3:**
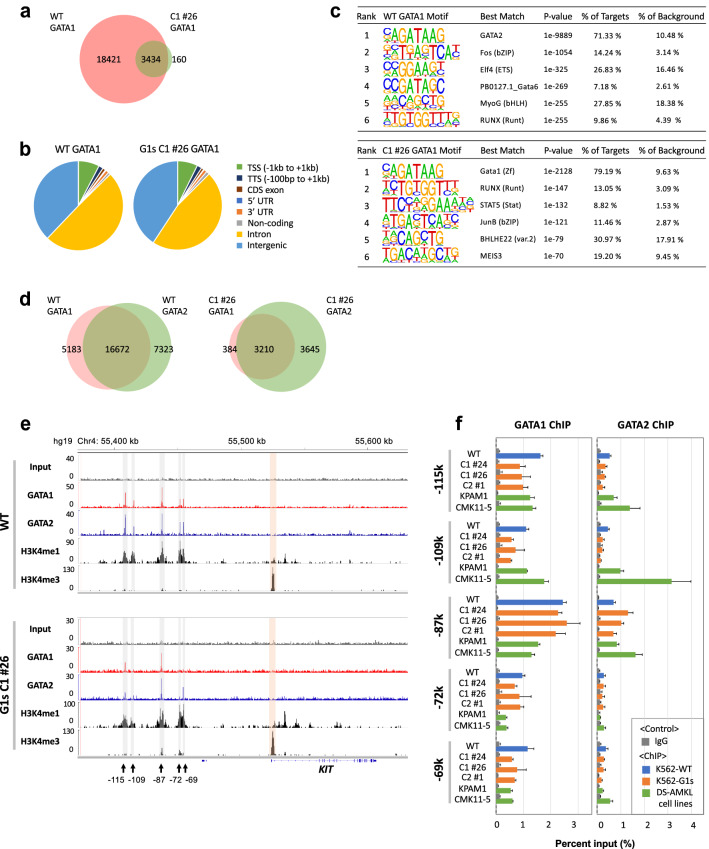
ChIP analysis of GATA1 and GATA2 in K562-WT and K562-G1s clones. (**a**) Overlap of peaks in GATA1 ChIP-seq of K562-WT and G1s C1 # 26. (**b**) Relative distribution of GATA1 ChIP-seq peak regions in the genome. (**c**) Results of de novo motif analysis of GATA1 ChIP-seq data. The top six motifs are shown. (**d**) Overlap of ChIP-seq peaks of GATA1 and GATA2 in K562-WT (left) and in G1s C1 #26 (right). Since both K562-WT and C1#26 had low yields of ChIP for GATA2, one library from each of the four experimental samples was prepared and sequenced. (**e**) Genome browser view at the *KIT* locus. The *y* axis shows read coverages normalized to RPGC (Read per genomic content). Light gray boxes indicate active distal regions (− 115 kb, − 109 kb, − 87 kb, − 72 kb and − 69 kb) marked with arrows, and the promoter region is highlighted in orange. The top shows profiles of K562-WT and the bottom shows profiles of the K562-G1s C1 #26. (**f**) Quantification of GATA1 and GATA2 binding upstream of *KIT* by ChIP-qPCR. (Left) data of GATA1 ChIP-qPCR, (right) data of GATA2 ChIP-qPCR. Results are averages of three independent experiments. Error bars represent standard deviation.

To determine why *KIT* expression is not suppressed in K562-G1s, we thought it would be important to test whether there is a change in GATA1 binding to the *KIT* locus. First, we performed ChIP-seq on K562-WT and K562-G1s C1 #26 to determine the location of GATA1 and GATA1s binding at the *KIT* locus. Western blotting showed that the anti-GATA1 ChIP antibody recognizes not only GATA1 but also GATA1s (Supplemental Fig. S3). In G1s C1 #26, although the number of GATA1 ChIP-seq peaks was lower than that of WT, 95.5% of the peaks overlapped with WT (Fig. 3a). Despite the difference in the number of peaks, the proportion of the peak regions of G1s C1 #26 distributed in the genome was almost the same as that of WT (Fig. 3b). The GATA binding motif was significantly enriched in both WT and in G1s C1 #26 (Fig. 3c). Since GATA1 shares GATA binding motif with GATA2, we also performed ChIP-seq of GATA2 (Fig. 3d). The percentage of GATA1 peaks overlapping with GATA2 was 76.3% in WT and 89.3% in G1s C1 #26, indicating that there are many regions where both GATA1 and GATA2 are functional even in G1s C1 #26. Compared to WT, G1s C1 #26 had a lower number of GATA2 and GATA1 ChIP-seq peaks and a lower ratio of the number of GATA1 ChIP-seq peaks to GATA2. For the *KIT* locus, GATA1 ChIP peaks were detected at − 115 kb, − 109 kb, − 87 kb, − 72 kb and − 69 kb of *KIT* in K562-WT, whereas the − 109 kb, − 72 kb and − 69 kb peaks were faint in G1s C1 #26 (Fig. 3e). In G1s C1 #26, GATA2 ChIP-seq showed a clear peak at − 87 kb as well as GATA1. Peaks at − 115 kb and − 69 kb were also detected, but the peak at -115 kb was faint. Thus, compared to K562-WT, neither GATA1s nor GATA2 binding positions were increased at the *KIT* locus in G1s C1 #26, but rather decreased.

To understand the status of the *KIT* gene of K562-WT and C1 #26 in more detail, we next performed Cleavage Under Targets & Release Using Nuclease **(**CUT&RUN)^34^-seq of histone modifications (Fig. 3e). The following observations were made in both cell lines; H3K4me1 modification, which is associated with gene enhancers, was mainly detected from − 115 to − 69 kb. H3K4me3, which is associated with gene promoters, was mainly detected around the transcription start site. Thus, the activation status of *KIT* demonstrated by the histone modifications was not significantly different between K562-WT and G1s C1#26.

Next, we quantified the binding of GATA1 and GATA2 by ChIP-qPCR using K562-WT, three K562-G1s clones; C1 #24, #26, C2 #1 and two ML-DS cell lines; KPAM1 and CMK11-5^35^ (Fig. 3f). In K562-WT and the K562-G1s clones, GATA1 and GATA2 were predominantly bound at -87 kb. Compared to K562-WT, GATA2 binding was not increased in the K562-G1s clones. In KPAM1 and CMK11-5, GATA2 bound more to − 115 kb and − 109 kb compared to K562-WT and K562-G1s clones, while GATA1 binding was comparable. These features may reflect differences in the open chromatin profiles and expression ratios of GATA1 and GATA2 in the cell lines. We also confirmed the binding of GATA1 and GATA2 to − 87 kb in these ML-DS cell lines. These data indicate that both GATA1 and GATA1s binds upstream of *KIT*, especially around -87 kb, commonly in K562-WT, the K562-G1s clones and the ML-DS cell lines. However, these results did not explain why *KIT* expression was suppressed in K562-WT and promoted in K562-G1s clones and the ML-DS cell lines.

### Enhancer/silencer assay of − 115 kb, − 109 kb, − 87 kb and + 1 kb region of ***KIT***

In the ChIP-seq experiments, five GATA1 binding sites were detected upstream of the *KIT* gene (Fig. 3e), but it was unclear which of these regions actually regulates the promoter of the *KIT* gene. Although the luciferase reporter assay cannot reproduce the entire regulatory mechanism of actual gene expression, it is possible to determine the potential strength of the gene expression regulatory function of the region of interest. Therefore, we attempted to assess enhancer activities of the GATA1 binding sites on the *KIT* locus. As the − 115 kb and − 87 kb regions had high ChIP signals (Fig. 3e–f), and the sequence of the − 109 kb region had homology to the − 114 kb enhancer region of mouse *kit*^32^ (Supplemental Fig. S4 and Table S2), we decided to assay these three regions.

DNase-seq data from the ENCODE project^36^ shows open chromatin profiles in a variety of cell types. In this data, for the upstream of *KIT*, obvious peaks were detected at the − 115, − 109, and − 87 kb regions in CMK and at the − 115 and − 87 kb regions in K562. Weak peaks were also present at the − 109, − 72, and − 69 kb regions of K562 (Supplemental Fig. S5a and Table S3). These peak positions are consistent with the binding positions of GATA1 and GATA2 in our ChIP assay (Fig. 3e–f). Furthermore, the DNase-seq data showed two peaks near the transcription start point of *KIT*; around the exon 1 and the **+ **1 kb region in intron 1 (Supplemental Fig. S5b). Interestingly, cell lines with high *KIT* expression (H1-hESC, CMK^37^, and HUVEC) tended to have high exon 1 peaks, and cell lines with lower expression (K562, HepG2, MCF7) tended to have higher **+ **1 kb peaks (Supplemental Table S4). These results suggest that the + 1 kb region might be involved in the suppression of *KIT* expression. Therefore, we included the **+ **1 kb region for further study.

**Figure 4:**
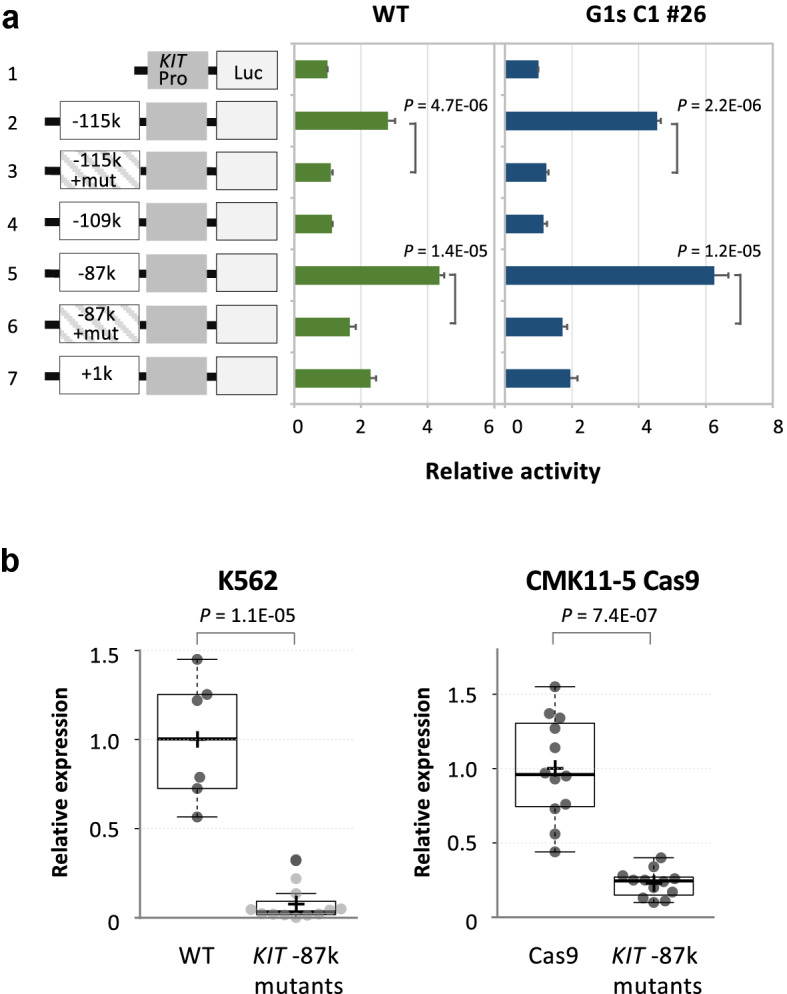
Enhancer/silencer assay of *KIT* and genome editing of GATA site in *KIT* − 87 k region. (**a**) Schematic diagram of reporter constructs and results of luciferase assay. The green solid bar indicates K562-WT and the blue bar K562-G1s C1 #26. The horizontal axis represents a relative activity with each value of the *KIT* promoter vector set at 1. The numbers on the vertical axis indicate the introduced vectors, whose schematic diagram are attached next to them. 1; *KIT* promoter vector, 2; − 115 kb, 3; − 115 kb with mutated GATA site, 4; − 109 kb, 5; − 87 kb, 6; − 87 kb with mutated GATA site, 7; **+ **1 kb region. Results are averages of three independent experiments. Error bars represent standard deviation. The *P*-values of the two-side t-test were given. (**b**) Expression levels of *KIT* in the *KIT* -87 kb GATA site mutants. Only the − 87 k homozygous mutants were selected for quantification of *KIT* expression. (Left) a comparison of six K562-WT single clones and twelve *KIT* − 87 kb mutants. Each sample mean is represented by a cross. Since most of the − 87 kb mutants were outside the quantitative range, their dots are shown in gray. (Right) twelve CMK11-5 Cas9 single clones and twelve *KIT* − 87 kb mutants. The vertical axis shows the relative value of the K562-WT single clones or CMK11-5 Cas9 single clones set to 1. The *P*-values of the Mann–Whitney U test were noted.

To determine if the sequences of the − 115 kb, − 109 kb, − 87 kb and **+ **1 kb regions of *KIT* possess enhancer or silencer activity in K562-WT and K562-G1s C1 #26, we conducted luciferase reporter assays with constructs of these regions connected to the *KIT* promoter fragment (Fig. 4a). Although the transcriptional activities were somewhat lower in K562-WT than G1s C1#26, the − 115 kb and − 87 kb regions showed enhancer activities in both K562-WT and G1s C1 #26. Introduction of a mutation in GATA binding motif (GATA > GAGC) of the − 115 kb or − 87 kb regions (Supplemental Fig. S4) significantly reduced enhancer activity (Fig. 4a), suggesting that these enhancer activities mainly came from factors binding to these GATA motifs. On the other hand, little enhancer activity of the -109 kb region was observed in both K562-WT and G1s C1 #26, and no silencer activity of the + 1 kb region was observed. These results showed that the DNA sequences in the − 115 kb and − 87 kb regions potentially have enhancer activity.

The − 87 kb region has the highest degree of GATA1 binding and transcriptional activity, suggesting that it plays the most important role in the regulation of *KIT* expression. To clarify how the GATA binding site in *KIT* − 87 kb contributes to *KIT* transcriptional regulation, we introduced the same GATA site mutation as above into the *KIT* − 87 kb region by genome editing using K562 and CMK11-5 Cas9, where Cas9 is constantly expressed to enhance genome editing efficiency (Supplemental Fig. S6). In K562-WT, *KIT* expression is repressed by GATA1, but in most of the K562 *KIT* − 87 kb mutant clones, *KIT* expression levels were further reduced and was below the quantitative range (Fig. 4b left panel). In the CMK11-5 Cas9 *KIT* − 87 kb mutant clones, *KIT* expression was reduced to one-fifth (*P* = 7.4E−07) (Fig. 4b right panel). These results demonstrated that the GATA site of *KIT* − 87 kb has an important function as an enhancer both in K562 and CMK11-5, and that *KIT* − 87 kb plays a major role in the transcriptional regulation of *KIT*, both in the presence of GATA1 and in the presence of GATA1s exclusively.

### GATA1s induces a different conformation of the *KIT* gene compared with GATA1

**Figure 5:**
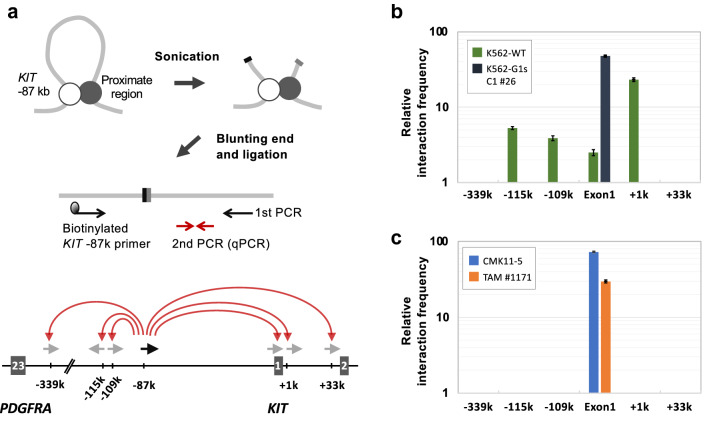
*KIT* gene conformation involving the *KIT* − 87 kb region. (**a**) Outline of the 3C-modified experiment. Upper left panel is an image of the *KIT* − 87 kb region and a conformationally proximate region. The white and black circles indicate transcription factor complexes containing GATA factors. Sonicated DNA–protein complexes (upper right) were end-blunted and ligated. Middle panel is a schematic diagram of PCR for detecting proximity with *KIT* − 87 kb region using the ligated DNA. Lower panel shows the positions of the primers used for the 1st PCR of this assay. The black arrow indicates the biotinylated *KIT* − 87 k primer and the gray arrows indicate the position of the test primers. The − 339 kb region is located 21 kb downstream of the *PDGFRA* gene, which is upstream of *KIT* gene. The + 33 kb region is located at intron1 of *KIT* gene. Both the − 339 kb and + 33 kb regions are used as the negative control regions. (**b**) Sonication-based 3C-assay of the *KIT* − 87 kb region with K562-WT and K562-G1s C1 #26. The horizontal axis represents the regions where the interaction was investigated. The vertical axis shows the relative value when the amplification amount of qPCR per 1 ng of each input DNA is set to 1. When the value was 1 or less, it was conceived that there was no effect of amplification by the first PCR, that is, the intended ligation product was not present, and the values were cut off. A representative data is shown. Error bars represent standard deviation. (**c**) Sonication-based 3C assay of *KIT* − 87 kb region with CMK11-5 and a TAM sample.

Although the results of the above ChIP and luciferase assays suggested the importance of the − 87 kb region in *KIT* expression, it was unclear how NTD deletion of GATA1 would increase *KIT* expression. Therefore, we decided to test in a 3C experiment whether the lack of the NTD of GATA1 causes differences in the interaction between the − 87 k region with the − 115 kb, − 109 kb, exon 1 and **+ **1 kb regions. However, conventional 3C or Capture-C derived from 3C uses restriction enzyme recognition sites^38–40^ that cannot distinguish exon 1 and the **+ **1 kb region with respect to the proximity of the − 87 kb region. In contrast, DNA fragmentation by sonication is possible with or without restriction enzyme recognition sites, and this treatment is expected to reduce noise by shaking off non-specific and weak peripheral chromatin interactions^41^. We therefore employed a sonication-based 3C assay^42^ (Fig. 5a). In brief, fixed genomic DNA was fragmented by sonication, blunt-ended and ligated. A first PCR was performed using the biotinylated *KIT* − 87 k primer and a primer designed to target the region of interest. The first PCR products purified by avidin magnetic beads were then used as a template for a second PCR (quantitative PCR).

In K562-WT, proximity of the − 87 kb region was detected with the − 115 kb, − 109 kb, exon 1 and + 1 kb regions (Fig. 5b). Since the value of exon 1 was 2.5 and that of + 1 kb region was 23.3, it was clear that most of the − 87 kb region interacted with the + 1 kb region rather than exon 1 in K562-WT. In contrast, in K562-G1s C1 # 26, proximity of the − 87 kb region was detected only with *KIT* exon 1 (Fig. 5b). To examine whether these findings would be relevant to DS-related myeloid disorders, we next performed 3C experiments with primary TAM cells as well as a ML-DS cell line, CMK11-5. A *GATA1* mutation (c.167_186del20) was confirmed in the TAM sample (Supplemental Fig. S7). Remarkably, in both CMK11-5 and this TAM sample, proximity of the − 87 kb region was detected only with *KIT* exon 1, just like K562-G1s C1 #26 (Fig. 5c). Thus, we found a difference in the three-dimensional structure of *KIT* between K562-WT, in which GATA1 is present, and K562-G1s C1#26, CMK11-5 and primary TAM cells, in which GATA1s is expressed in place of GATA1. These results demonstrated that lack of GATA1 NTD introduced a conformation change of *KIT* and suggests that this change led to dysregulation of *KIT*. Regulation of gene conformation is a novel property of GATA1 NTD and may be a mechanism involved in TAM blast proliferation.

## Discussion

K562-WT cells, like normal megakaryocyte-erythroid progenitor (MEP) cells, express the full-length GATA1 protein (GATA1FL) and a small amount of GATA1s (Fig. 1b). Therefore, we actually compared the binding of GATA1s and (GATA1FL + GATA1s) to the *KIT* gene and its regulation. Gialesaki et al.^43^ also reported decreased expression of the common erythroid markers CD235a and CD71 in their GATA1s-K562 cells. However, the severe decrease in proliferation seen in their GATA1s-K562 cells was not observed in our K562-G1s cells. Our K562-G1s cells enabled us to investigate the function of the NTD of GATA1 and a possible mechanism of TAM development through acquisition of *GATA1* mutation in DS.

We performed ChIP-seq and identified the location of GATA1 binding sites upstream of *KIT* in K562-WT and a K562-G1s C1 #26. ChIP-qPCR analysis confirmed that the occupancy in the K562-G1s clones tended to be lower than that of K562-WT except for the − 87 kb region (Fig. 3d). As GATA1s retains the two Zn fingers involved in DNA binding, the low occupancy of GATA1s is interesting. Due to the lack of NTD, the affinity of protein-DNA complexes formed by GATA1s may be weaker than that of GATA1. It has been reported that GATA1s binds to many of its erythroid-specific target genes less efficiently than full length GATA1^17,44^. However, since *KIT* is not an erythroid-specific gene, their findings may not be applicable to our results*.* In K562-G1s clones, binding of GATA2 at the *KIT* locus was also lower than that of K562-WT except at the − 87 kb region (Fig. 3d). Therefore, it is likely that the open chromatin pattern of the K562-G1s clones is partially different from that of K562-WT. However, the partial low binding of GATA1s and GATA2 to *KIT* in K562-G1s clones cannot explain the increased expression of *KIT*.

Jing et al.^32^ reported on the transcriptional regulation of *kit* by Gata factors in a mouse experimental system. Their model is interesting; in immature erythrocytes, prior to Gata1 expression, Gata2 binds to the − 114 kb *kit* distal enhancer, which is in physical proximity to the *kit* active promoter. When Gata1 is expressed along with cell maturation, Gata1 displaces Gata2, a processed termed “GATA switching”, and triggers a loss of the enhancer/promoter interaction. In contrast to the mouse experimental system, both GATA1 (or GATA1s) and GATA2 are simultaneously expressed in K562-WT, K562-G1s clones, TAM and ML-DS blast cells^1^. In K562-G1s clones expressing GATA1s exclusively, the occupancy of GATA2 upstream of *KIT* was not increased compared to K562-WT (Fig. 3e,f), suggesting that the elevated expression of *KIT* is not due to “GATA switching”. Previous study has shown that GATA2 is an important activator of *KIT* transcription^32^. However, siRNA knockdown experiments of *GATA2* did not change the expression of *KIT* in K562 cells. Although these results suggest that the contribution of GATA2 to the expression of *KIT* in K562 cells is not large, they do not rule out the involvement of GATA2 in the expression of *KIT* at the basal level in K562 cells. Continued expression of GATA2 may affect *KIT* expression in K562-G1s cells. Even in the studies which comprehensively identified factors that interact with GATA1 by mass spectrometry, GATA2 was not identified as a factor that binds to GATA1^45,46^. Therefore, it is likely that GATA1 and GATA2 do not bind to the same locus at the same time, but rather in different cells. Given that GATA2 does not contribute significantly to *KIT* expression in K562 cells, GATA1 may play a central role in the formation of the 3C configuration and transcriptional regulation of *KIT*.

Physical interactions between genomic regions play important roles in the regulation of genome function, such as gene expression^47^. In this study, sonication-based 3C assay showed that lack of GATA1 NTD altered the genomic structure of *KIT*. Our present data indicate that in the presence of GATA1, the − 87 kb region gathers the − 115 kb and − 109 kb regions and interacts with the **+ **1 kb region more than exon 1 (promoter), resulting in suppression of *KIT* expression. However, when GATA1 is replaced to GATA1s, the − 87 kb region interacts only with exon 1, leading to *KIT* expression induction. Thus, the − 87 kb region may function as a hub to regulate *KIT* gene expression in the presence of GATA1. These findings raise two important questions. Firstly, what is the functional mechanism for suppression of *KIT* expression caused by the genomic conformation comprising of the assembly of *KIT* − 115 kb, − 87 kb, and − 109 kb regions into the **+ **1 kb region? We assume that such genomic conformation will lead to collective isolation of the active enhancers with POLR2A from the promoter. Second, how does the NTD of GATA1 control genomic conformation of *KIT*? So far, it has been reported that Rb1^48^ and E2F1^49^ binds to the N terminus of GATA1. Although they are potential GATA1 NTD partners, it is unlikely that they have functions involved in loop formation. Interaction of another factor, such as CTCF, which regulates chromatin structure, may be more likely. Interaction between CTCF and GATA1 has been reported^50^. The NTD of GATA1 may be leading the *KIT* enhancer to the + 1 kb region by combining with CTCF. It is interesting that the CTCF and E2F recognition motifs are present in the + 1 kb site (Supplemental Fig. S8).

*MYC*, *GATA2* and *KIT* are the genes that Bourquin et al.^25^ described as highly expressed in ML-DS compared with non-DS-AMKL. While all three genes are repressed by GATA1, the mechanisms of dysregulation by GATA1s do not appear to be same. Maroz et al.^51^ has previously shown that GATA1s has an impaired ability to repress oncogenic MYC that is likely due to a reduced occupancy at the *MYC* promoter. In their study, they showed that GATA1s occupancy at the *MYC* promoter was reduced in CMK cells. Ling et al.^52^ reported that Gata2 is overexpressed in Gata1s erythroid cells accompanied by a marked reduction of H3K27me3 in the regulatory element of the *Gata2* gene. The effects of the GATA1 NTD deletion on the regulation of gene expression appear to be complex.

In summary, we clearly showed that NTD loss of GATA1 resulted in significantly higher expression of *KIT* compared to K562-WT, switching to an exclusive interaction between the − 87 kb region and exon 1 of the *KIT* gene, which was also observed in the ML-DS cell line CMK11-5 and primary TAM cells. However, to more accurately assess how the GATA1 NTD regulates genomic conformation and gene expression of *KIT*, it seems necessary to generate a K562 cell line expressing only GATA1FL and test it against K562*-*G1s.

## Methods

### Cell lines and a TAM sample

CMK11-5, a subclone of CMK, and KPAM1 cells are established from patients with ML-DS. K562, a chronic myelogenous leukemia cell line was purchased from JCRB Cell Bank. K562 and CMK11-5 were cultured with RPMI1640 containing 10% fetal bovine serum. KPAM1 was cultured by adding SCF (NKMAX) at 100 ng/mL to the above conditions. To improve the low efficiency of CRISPR genome editing in CMK11-5 cells, we established CMK11-5 Cas9 cells that constantly express Cas9. Details are provided in the Supplemental Methods online*.* Peripheral blood was obtained from a 1-day-old male TAM patient (#1171) after informed consent. The white blood cell count was 151,200/µl with 84% of blast cells. The method for detecting a *GATA1* mutation in the blast cells is as described previously^53^. Ficoll-Hypaque-separated cells were resuspended in CELLBANKER (ZENOAQ RESOURCE) and stored at − 80 °C until use. The cells were thawed and incubated in STEM SPAN SFEM (STEMCELL Technologies) supplemented with 100 ng/ml of SCF for three days. This study was approved by the Ethics Committee of Hirosaki University. All experiments were performed in accordance with the principles of the Declaration of Helsinki.

### CRISPR genome editing

Target sequences and a single-stranded oligonucleotide (ssODN) sequence are listed in the Supplemental Table S5. For genome editing of *GATA1*, annealed DNA oligos that code for the target-specific crRNA were ligated into GeneArt CRISPR Nuclease Vector Kit (Thermo Fisher Scientific). 20 µg of the CRISPR vector plasmid DNA was transfected to 1 × 10^6^ cell of K562 cells with 4D-Nucleofector (Lonza). After 72 h, the cells were subjected to limiting dilution. To introduce a mutation (GATA > GAGC) at the *KIT* − 87 kb GATA site via homology-directed repair (HDR), 120 pmol of KIT − 87 k crRNA:tracrRNA duplex complexed with 104 pmol of Cas9 Nuclease V3 (Integrated DNA Technologies) and 100 pmol of KIT − 87 k ssODN were transfected into 2 × 10^5^ K562 cells by 4D-Nucleofector. After 5 days, the cells were subjected to limiting dilution. For genome editing in CMK11-5 Cas9 cells, 100 pmol of KIT − 87 k crRNA:tracrRNA duplex and 100 pmol of KIT − 87 k ssODN were transfected by 4D-Nucleofector. During this time, the cells were cultured using Maurissen and Woltjen's method^54^ to increase the efficiency of HDR. In detail, uncloned bulk CMK11-5 Cas9 cells were pretreated with 2 mM NU7441 (TOCRIS) and 1 mM SCR7 (Xcess Bioscience) for 4 h before and treated for 48 h after transfection. 33 mM XL413 (Sigma-Aldrich) was also added after transfection and incubated for 24 h. The cells were cold-shocked by incubation at 32 °C for 48 h after transfection. After 7 days, the cells were subjected to limiting dilution.

### RNA-seq and qRT-PCR

RNA was extracted by RNeasy mini plus kit (QIAGEN). mRNA selection was performed using NEBNext Poly(A) mRNA Magnetic Isolation Module (New England Biolabs). Library preparation for RNA-Seq analysis was conducted with the KAPA RNA HyperPrep Kit (KAPA BIOSYSTEMS). RNA-seq libraries were sequenced on the Illumina NextSeq 550. Sequence reads were aligned to the GENCODE human reference genome GRCh38 using STAR^55^ (v. 2.6.1d). Aligned reads were counted by RSEM^56^ (v1.3.1). The count data normalized using the DESeq2 package^57^ (v1.32.0) were subjected to GSEA^31^. cDNA was synthesized using iScript reverse transcription supermix for RT-qPCR (Bio-Rad Laboratories). qRT-PCR was performed with iQ SYBR green supermix kit and the CFX96 Real-time PCR system (Bio-Rad Laboratories). All primers used in this paper are listed in the Supplemental Table S6.

### GATA1 knockdown with siRNA

A *GATA1* siRNA was designed according to the guideline of Japan Bio Services. The sequence is as follows. hGATA1-2: 5′-ACUGACAAUCAGGCGCUUCTT-3′. siRNAs purchased from Thermo Fisher Scientific Dharmacon were ON-TARGET plus SMART pool human GATA1 (L-009610-00-0005), GATA2 (L-009024-00-0005) and a non-targeting pool (D-001810-10-20). To knockdown of *GATA1* or *GATA2* expression, cells were transfected by using 4D-Nucleofector with 1 µL of 40 nmol/l siRNA solutions per 4 × 10^5^ cells. After 48 h, the cells were collected and total RNA and whole cell lysate were extracted.

### ChIP and CUT&RUN assays

Chromatin samples were prepared using SimpleChIP Enzymatic Chromatin IP Kit (Cell signaling technology) according to the manufacturer's protocol. ChIP assays were performed with anti-GATA1 (ab11852, Abcam) and GATA2 (H-116, Santa Cruz) antibody. Quantitative PCR was performed with KAPA SYBR FAST qPCR MasterMix (KAPA BIOSYSTEMS) on some of the regions where peaks were observed in ChIP-seq. Primers used in the analysis are shown in Supplemental Table S6. For sample preparations of ChIP-seq, Dynabeads anti-rabbit IgG (Thermo Fisher Scientific) was used to reduce noise instead of the Protein G magnetic beads included in the kit. Library preparation was conducted with the KAPA HyperPrep Kit (KAPA BIOSYSTEMS). ChIP-seq libraries were sequenced on the Illumina NextSeq 550 (1 × 75 bp). Sequence reads were aligned to the UCSC human genome assembly hg19 using Bowtie2^58^ (ver. 2.2.2) with option –N1. Duplicated reads were removed by Picard v2.18 software (Broad Institute). For visualization, BAM files were converted to bigWig coverage files using bamCoverage command of deepTools^59^ (v3.1.3). Read coverages were normalized to RPGC (Read per genomic content). For GATA1 and GATA2 ChIP-seq data, peak calling, identification of overlapping peaks, and comprehensive motif analysis were performed by HOMER^60^ (v4.11) findPeaks with -style factor option, mergePeaks, and findMotifsGenome.pl, respectively.

CUT&RUN assays were performed with CUT&RUN Assay kit (Cell Signaling Technology) according to the manufacturer's protocol. The antibodies used were anti-H3K4me1 (39298, Active Motif) and H3K4me3 (9751, Cell Signaling Technology). Libraries were size selected for 150–350 bp range with KAPA Pure Beads (KAPA BIOSYSTEMS). Samples were sequenced on the Illumina MiSeq (2 × 75 bp). Sequence reads were aligned to the UCSC human genome assembly hg19 using bowtie2 with the same settings as described in the paper by Skene and Henikoff^61^. Duplicated reads were removed by Picard (Broad Institute).

### Enhancer assays

Each region of the *KIT* locus was amplified by PCR. Used primers were listed in Supplemental Table S6. PICA KIT pro vector was constructed by embedding of about 160 bp of *KIT* promoter region^62^ into SmaI-BglII site of PICA gene basic vector (Toyo Ink). Each fragment of *KIT* − 115 kb, − 109 kb, − 87 kb and **+ **1 kb region were ligated to SmaI sites of the PICA KIT pro vector. Mutated GATA site (GATA > GAGC) was induced by inverse PCR. K562-WT or a K562-G1s C1 #26 cells were seeded at a density of 4 × 10^5^ cells/well (24 well plate). The cells were transfected using lipofectamine 2000 reagent (Thermo Fisher Scientific) with 800 ng of luciferase reporter and 2 ng of pGL4.74 [hRluc/TK] Renilla luciferase expression vector (Promega) as an internal control. The cells were harvested 24 h after transfection, and cell extracts were assayed using the Dual-Luciferase reporter assay kit (Promega).

### Sonication-based 3C assay

Experiments were performed according to the method of Fujita et al.^42^ with some changes. Briefly, formaldehyde was added to 1 × 10^7^ K562 cell culture at a final concentration of 0.35%, and to CMK11-5 and TAM blast cell culture at a final concentration of 0.3%, respectively, and fixed for 5 min. The chromatin was extracted and fragmented by sonication (at an average fragment length of 1.2 kb) with UD-200 BIORUPTOR (Cosmo Bio), and short fragments were subsequently removed by CHROMA SPIN TE-1000 columns (Clontech-Takara Bio). Size selected chromatin samples were treated with the End-It DNA End-Repair kit (Lucigen) at room temperature for 45 min. After heating at 70 °C for 10 min, the reaction mixture was incubated in the presence or absence of T4 DNA ligase (Roche Diagnostics, Mannheim, Germany) at room temperature for 4 h. After reverse cross-linking, sample DNA was purified by phenol/CIA extraction and ethanol precipitation. For the first PCR, a 5' end -biotinylated common primer was prepared, which contained the I-SceI site and *KIT* − 87 kb site. In addition, primers were designed in regions where interaction with *KIT* − 87 kb region was to be examined. KOD FX Neo (Toyobo) was used for the first PCR. The PCR products were size-selected by 0.6 × Agencourt AMPure XP Beads (Beckman Coulter). The samples were selected with Dynabeads M-280 Streptavidin (Thermo Fisher Scientific) and 500 µl of RIPA buffer (50 mM tris(hydroxymethyl)aminomethane [pH 7.5], 150 mM NaCl, 1 mM EDTA, 0.5% sodium deoxycholate, 0.1% SDS, 1% IGEPAL-CA630) at 4 °C for 1 h. After three washes with RIPA buffer and one wash with 1 × Cut Smart buffer (New England Biolabs), the Dynabeads were treated with I-SceI (New England Biolabs) at 37 °C for 2 h. The supernatant was head-inactivated at 65 °C for 20 min and used for the second qPCR with KAPA SYBR FAST qPCR MasterMix. Primers used in the analysis are shown in Supplemental Table S6.

### Statistical analysis

All statistical tests were performed using the EZR (R-statistics) package^63^ (v1.54) with a significance level of 0.05. After confirming the normality with Shapiro–Wilk normality test, t-test was performed. Mann–Whitney U test was used to compare sample groups for which normality was rejected.

## Supplementary Information

Supplementary Information.

## Data Availability

All RNA-seq, ChIP-seq and CUT&RUN data in this manuscript are available at GEO under accession number GSE159072.
