# Supplementary material for: Mechanism of KIT gene regulation by GATA1 lacking the N-terminal domain in Down syndrome–related myeloid disorders
Source: Sci Rep. 2022 Nov 29;12:20587. doi: 10.1038/s41598-022-25046-z (PMC9708825; doi:10.1038/s41598-022-25046-z)
Supplement: Supplementary file 1 — Supplementary Information. [file 41598_2022_25046_MOESM1_ESM.doc]

**Supplementary Information**

**Mechanism of *KIT* gene regulation by GATA1 lacking the N-terminal domain in Down syndrome–related myeloid disorders**

Rika Kanezaki,^1^ Tsutomu Toki,^1^ Kiminori Terui,^1^ Tomohiko Sato,^1^ Akie Kobayashi,^1^ Ko Kudo,^1^ Takuya Kamio,^1^ Shinya Sasaki,^1^ Koji Kawaguchi,^2^ Kenichiro Watanabe,^2^ and Etsuro Ito^1, 3, *^

^1^Department of Pediatrics, Hirosaki University Graduate School of Medicine, Hirosaki, Japan

^2^Department of Hematology and Oncology, Shizuoka Children's Hospital, Shizuoka, Japan.

^3^Department of Community Medicine, Hirosaki University Graduate School of Medicine, Hirosaki, Japan

*Corresponding author: Etsuro Ito - eturou@hirosaki-u.ac.jp

Contents: Supplemental Tables 1-6, Figures 1-9, Methods and References.

**Supplemental Table 1: GATA1 mutations in K562-G1s clones.**

**
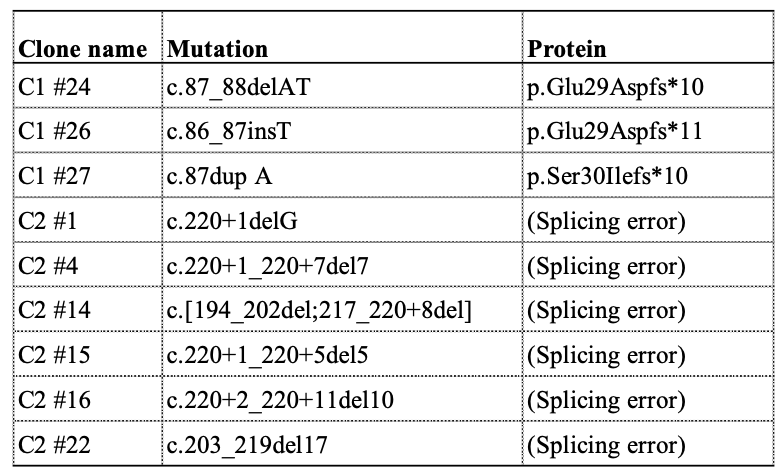
**

**Supplemental Table 2: Details of the databases cited in Supplemental Figure 4.**

**
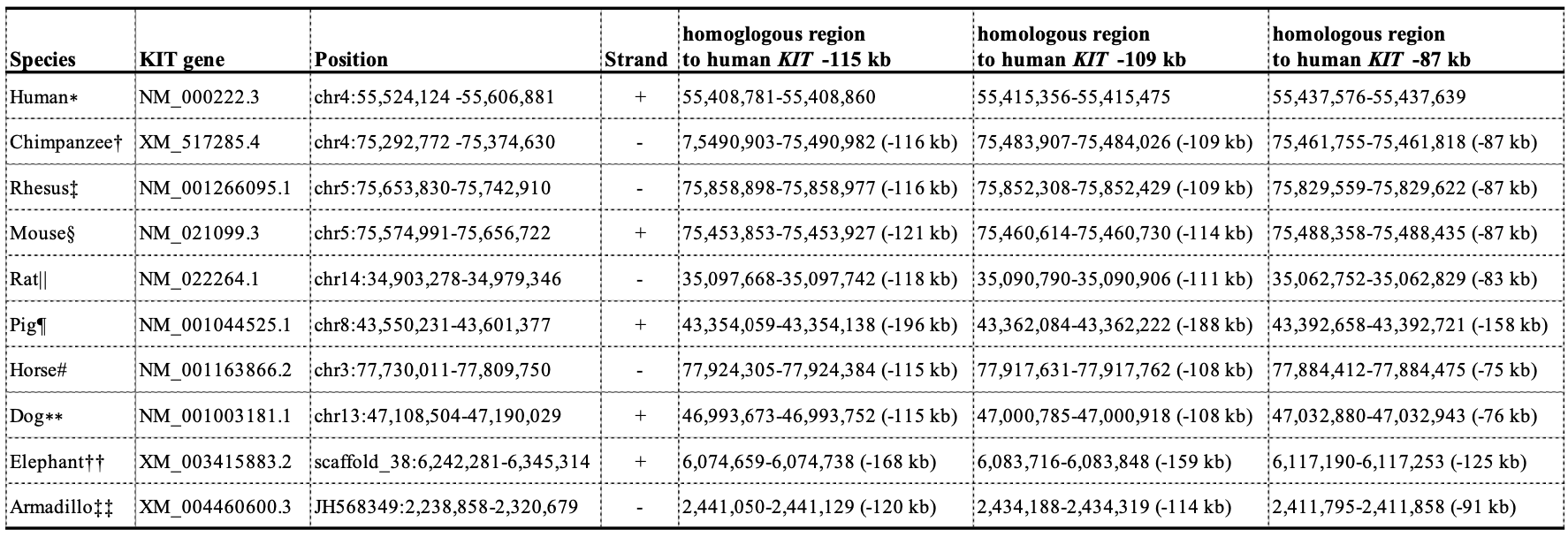
**

The following databases were used as references; ∗ GRCh37/hg19 (Feb, 2009), † CSAC 2.1.4//panTro4 (Feb, 2011), ‡ BGI CR_1.0/rheMac3 (Oct, 2010), § GRCm38/mm10 (Dec, 2011), || RGSC 5.0/rn5 (Mar, 2012), ¶ SGSC Sscrofa10.2/susScr3 (Aug, 2011), # Broad/equCab2 (Sep, 2007), ∗∗ Broad CanFam3.1/canFam3 (Sep, 2011), †† Broad/loxAfr3 (Jul, 2009), ‡‡ Baylor/dasNov3 (Dec, 2011).

The numbers in (kb) indicate the distance from the transcription start site of the *KIT* gene in each species to the homologous region to human *KIT* -115, -109 and -87 kb region.

**Supplemental Table 3: ENCODE data used in Supplemental Figure 5.**


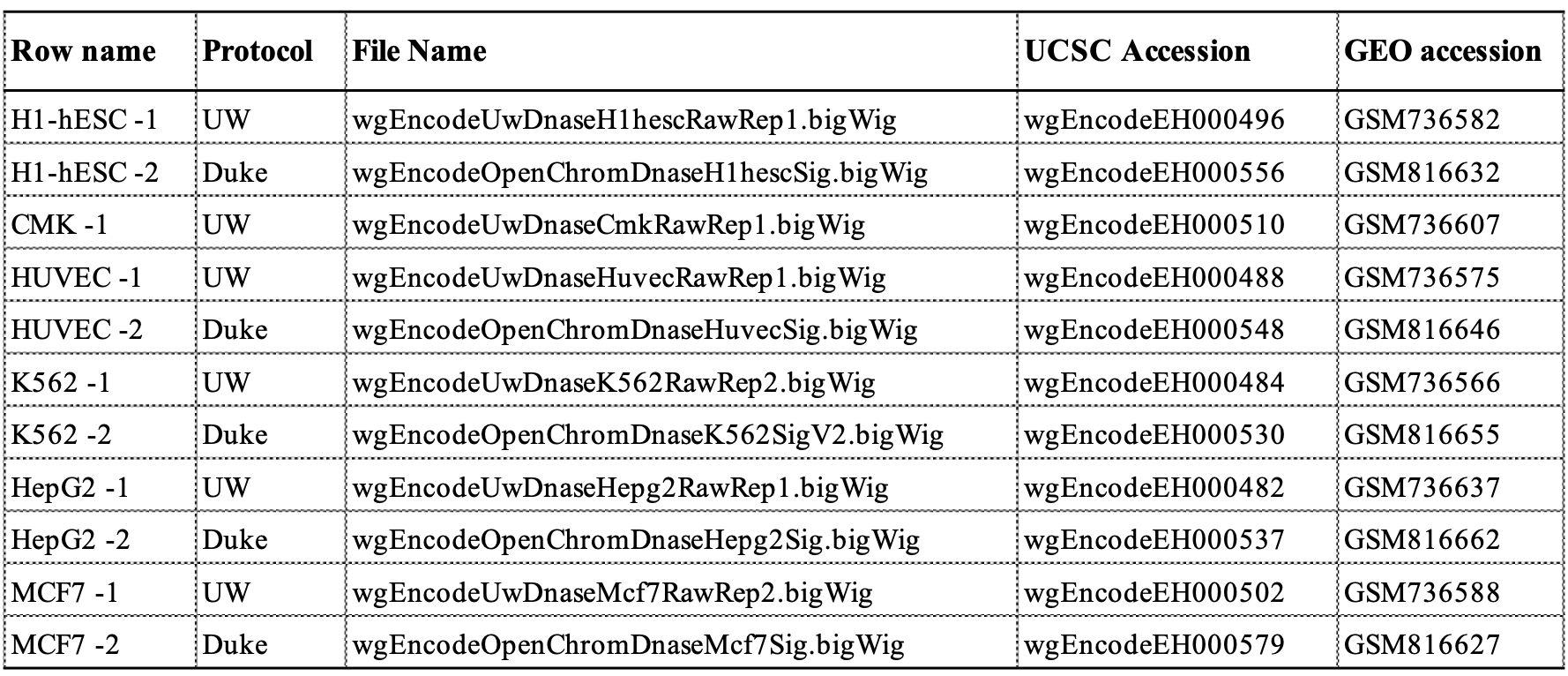


The files were obtained from the ENCODE repository at UCSC.

**Supplemental Table 4: Expression levels of *KIT* in RNA-seq from ENCODE/Caltech in FPKM.**


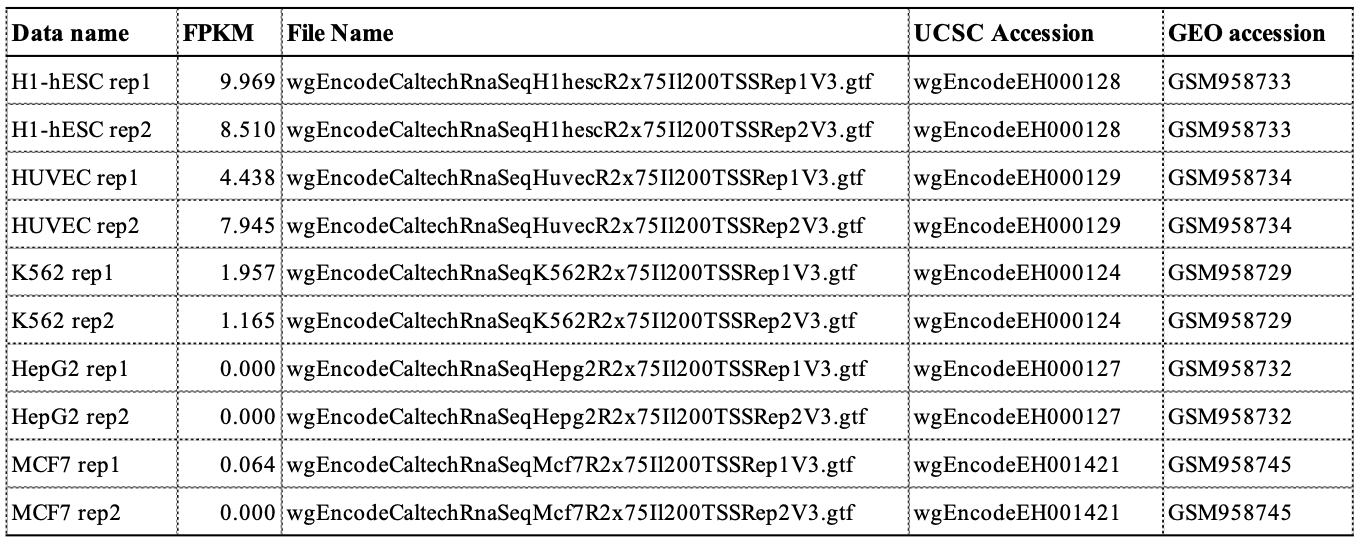


FPKM; fragments per kilobase of exon per million reads mapped.

The files were obtained from the ENCODE repository at UCSC.

The expression estimates for GENCODE GRCh37.v3c transcription start sites (TSS) of the *KIT* gene were extracted from the files above.

There are no RNA-seq data of CMK in the ENCODE repository at UCSC.

**Supplemental Table 5: Target sequences and ssODN sequence for genome editing.**


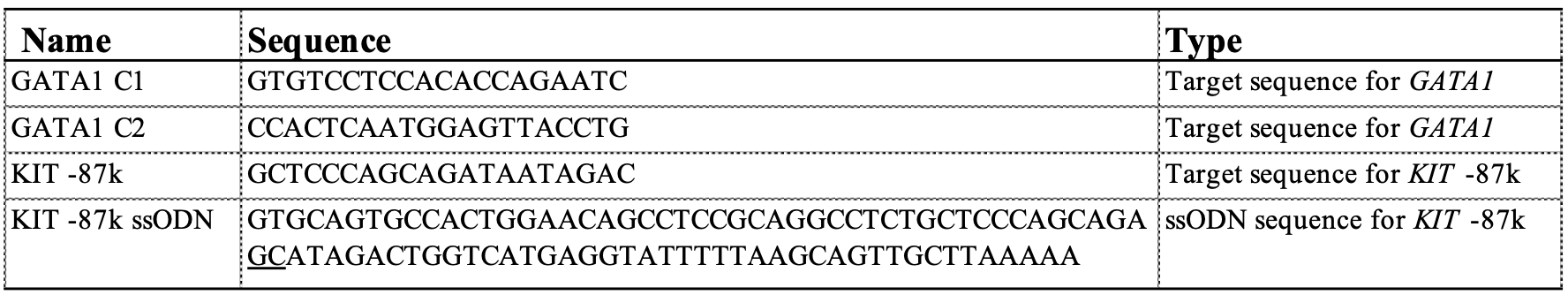


ssODN; single-stranded oligonucleotide.

Target sequences were designed by Zhang Lab’s gRNA design tool (https://zlab.bio/guide- design-resources).

The underline in the Sequence of KIT -87k ssODN indicates the mutated bases in the GATA binding site.

**Supplemental Table 6: Primers used in this paper.**


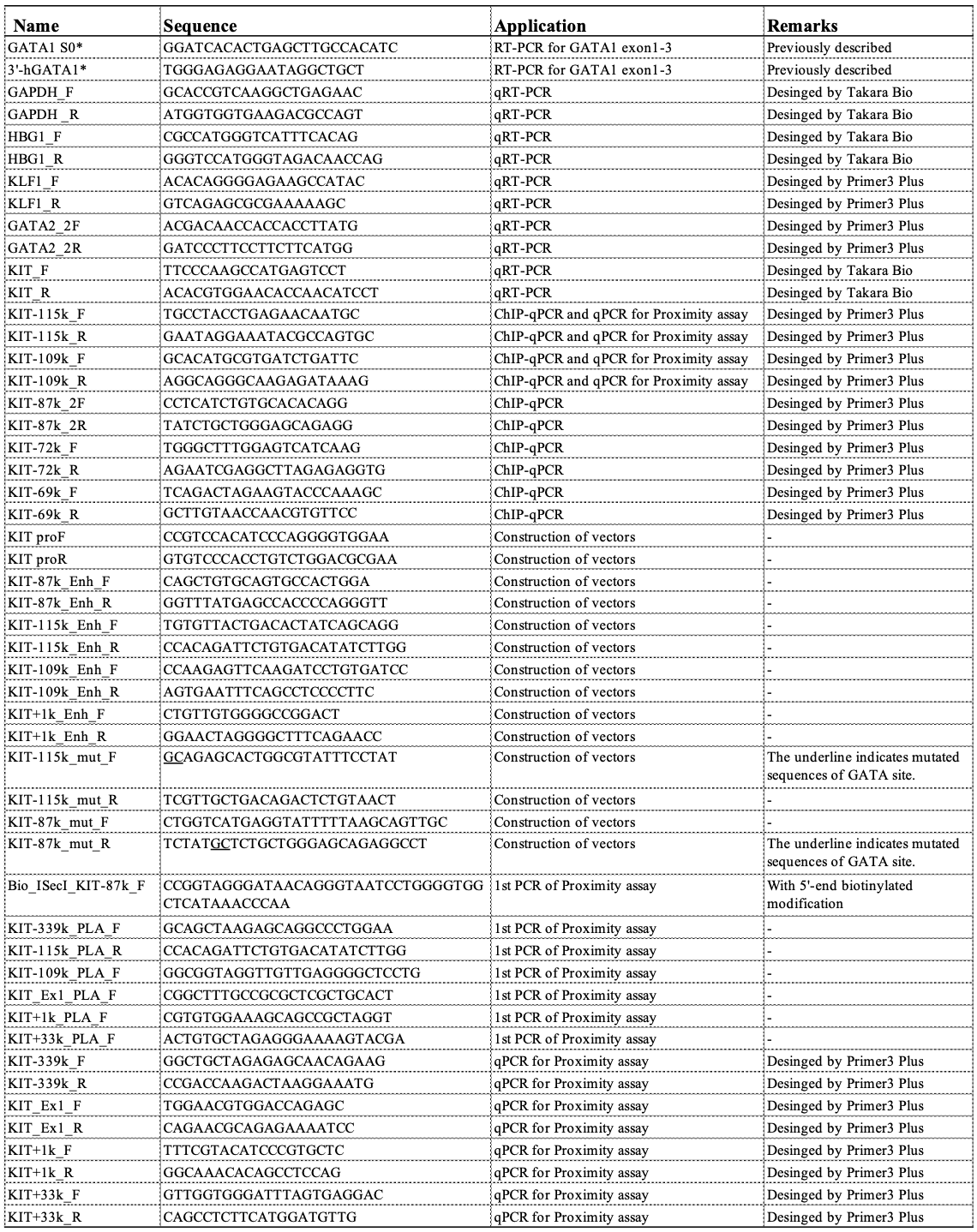


*These primers are used in the following reference; Terui, K. *et al*. Highly sensitive detection of GATA1 mutations in patients with myeloid leukemia associated with Down syndrome by combining Sanger and targeted next generation sequencing. *Genes Chromosomes Cancer*. **59**, 160-167 (2020).

Primers for qPCR analysis were designed by Primer3 Plus (http://www.bioinformatics.nl/cgi-bin/ primer3plus/ primer3plus.cgi/) or the Perfect Real Time Support System for Array (Takara Bio).

**
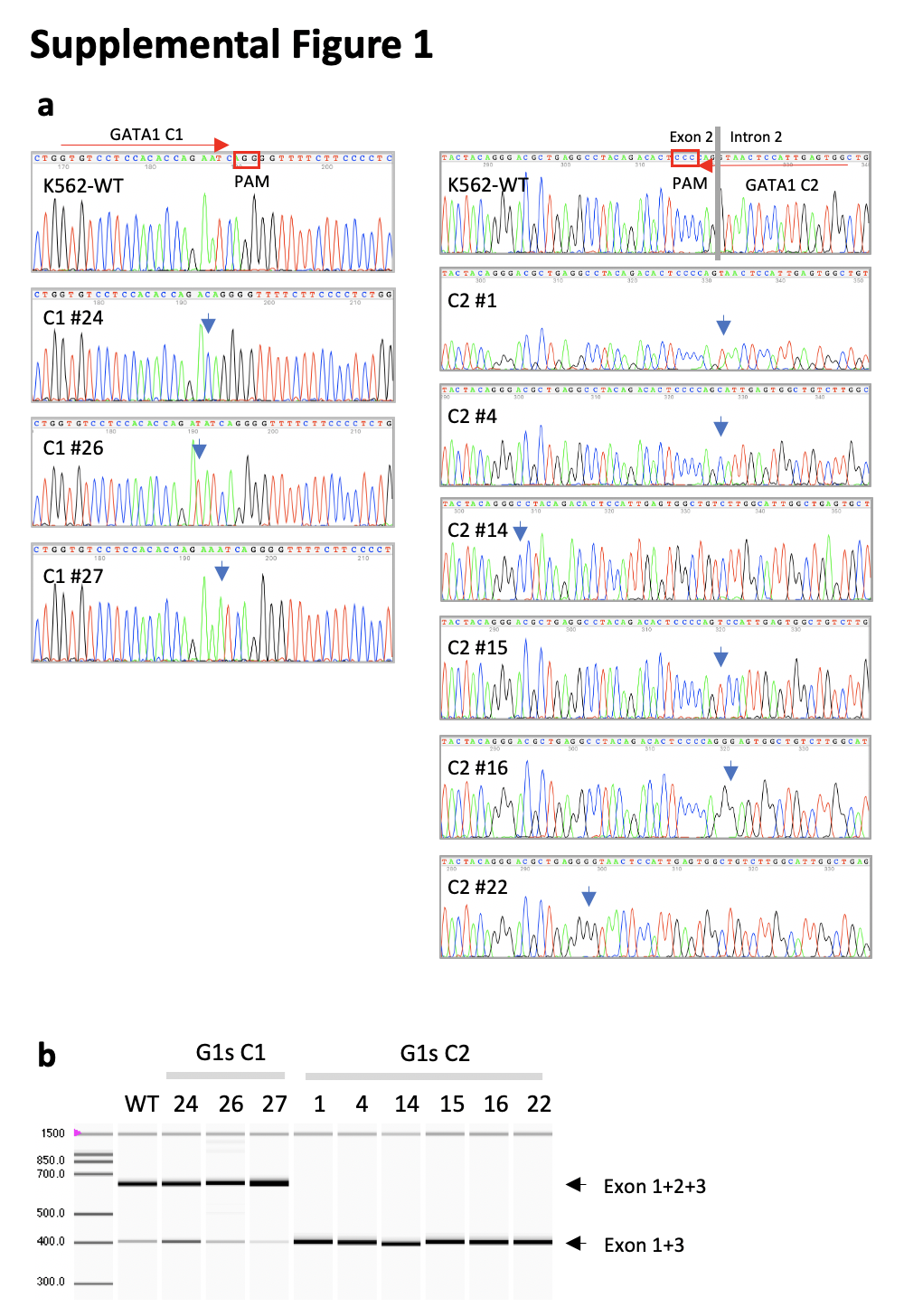
**

**Supplemental Figure 1. Validations of *GATA1* mutation in the K562 G1s clones. (**a) Sequencing of *GATA1* genomic DNA in K562-G1s clones. The blue arrows indicate the start position of each mutation. The red arrows indicate the target sequences for each genome editing. PAM: proto-spacer adjacent motif. (b) Splicing test of *GATA1* exon 2. RT-PCR was performed with primers placed at exon 1 and exon 3 of *GATA1*. PCR products were run on an automated electrophoresis system (Experion: Bio-Rad). If exon 2 of *GATA1* mRNA is spliced out, the PCR product will be 239 bp shorter. PCR products containing the exon 2 are not detected in K562-G1s C2 clones. Primers used in this test are listed in Supplemental Table S6.

**
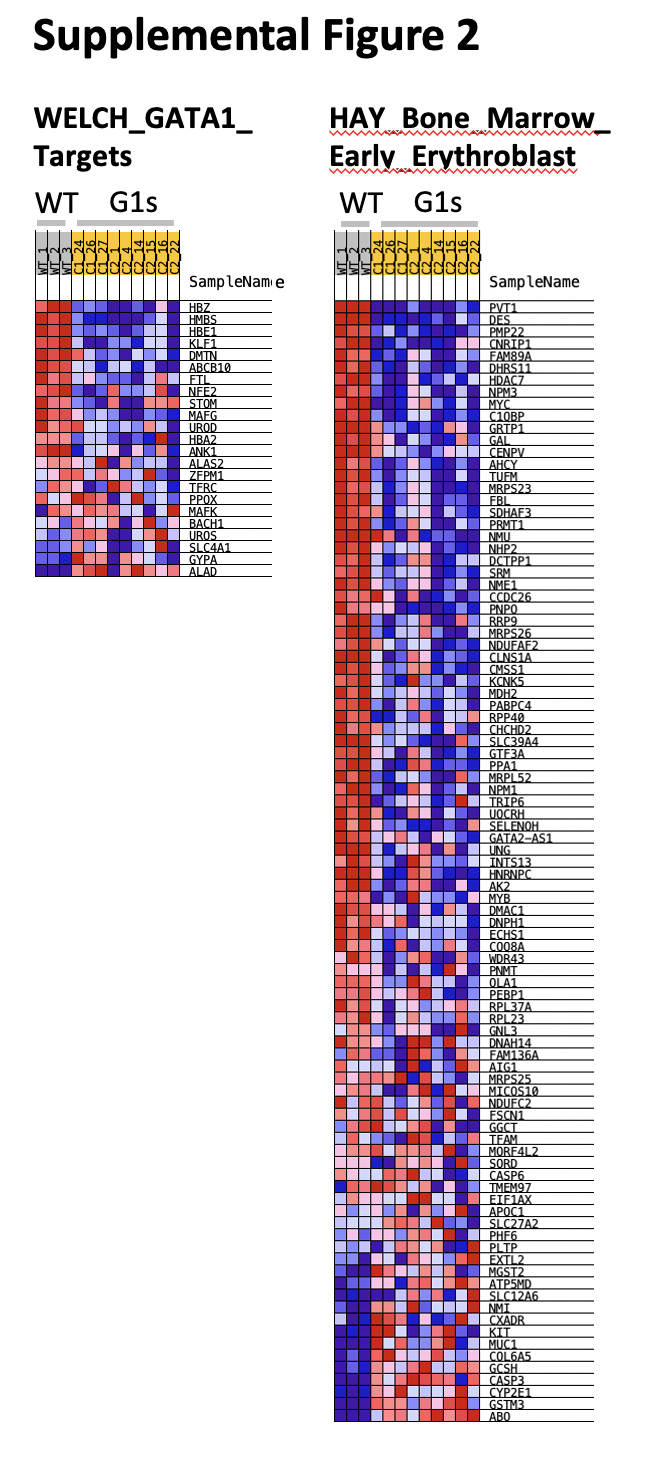
**

**Supplemental Figure 2. Expression profile of the K562 G1s clones.** Heat maps of the gene sets discussed in Fig. 1c. (Left) Gene Set: WELCH_GATA1_Targets. (Right) Gene Set: HAY_Bone_Marrow_Early_Erythroblast.

**
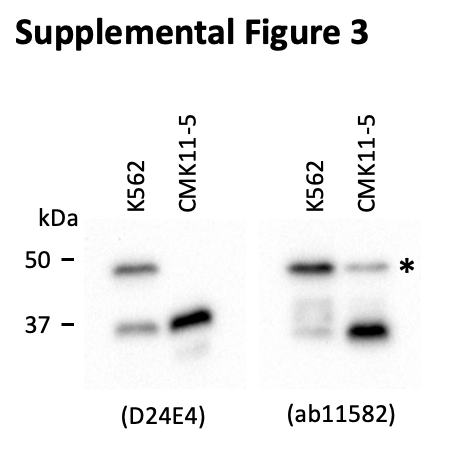
**

**Supplemental Figure 3. Anti-GATA1 (ab11582) antibody used in our GATA1 ChIP experiments recognizes GATA1s protein.** Western blotting was performed with K562 WT and CMK11-5. The anti-GATA1 (D24E4) antibody was used as a control since it recognizes both GATA1 and GATA1s proteins (see Fig. 1b). Images were obtained using ChemiDoc MP (Bio-Rad Laboratories). *: Nonspecific signal.

**
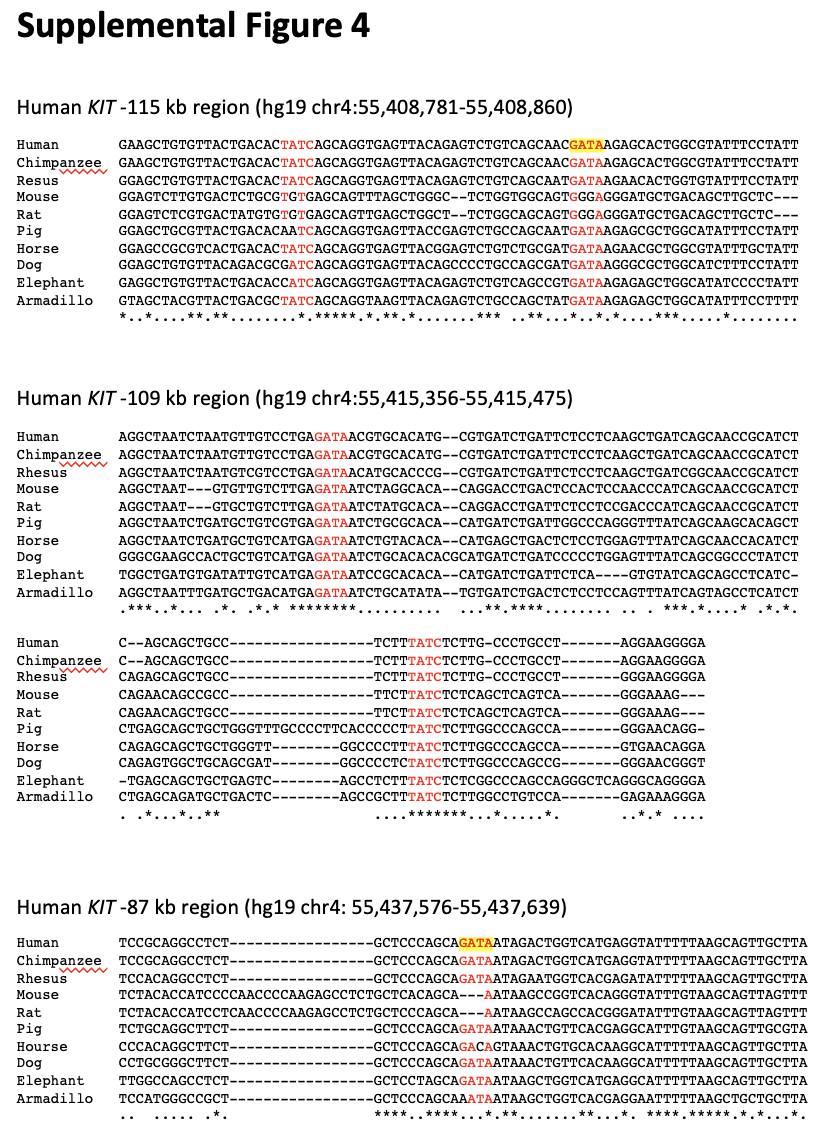
**

**Supplemental Figure 4. Conservation of *KIT* -115k, -109k and -87k regions.** The core sequences of human *KIT* -115kb, -109 kb, and -87 kb regions were compared with genomic sequences of other species. Homology with sequences in these regions was found only in mammals. The Red letters indicate GATA sites. The GATA sites in the yellow background are the positions where the mutation (GATA>GAGC) was introduced in the enhancer/silencer assay.

**
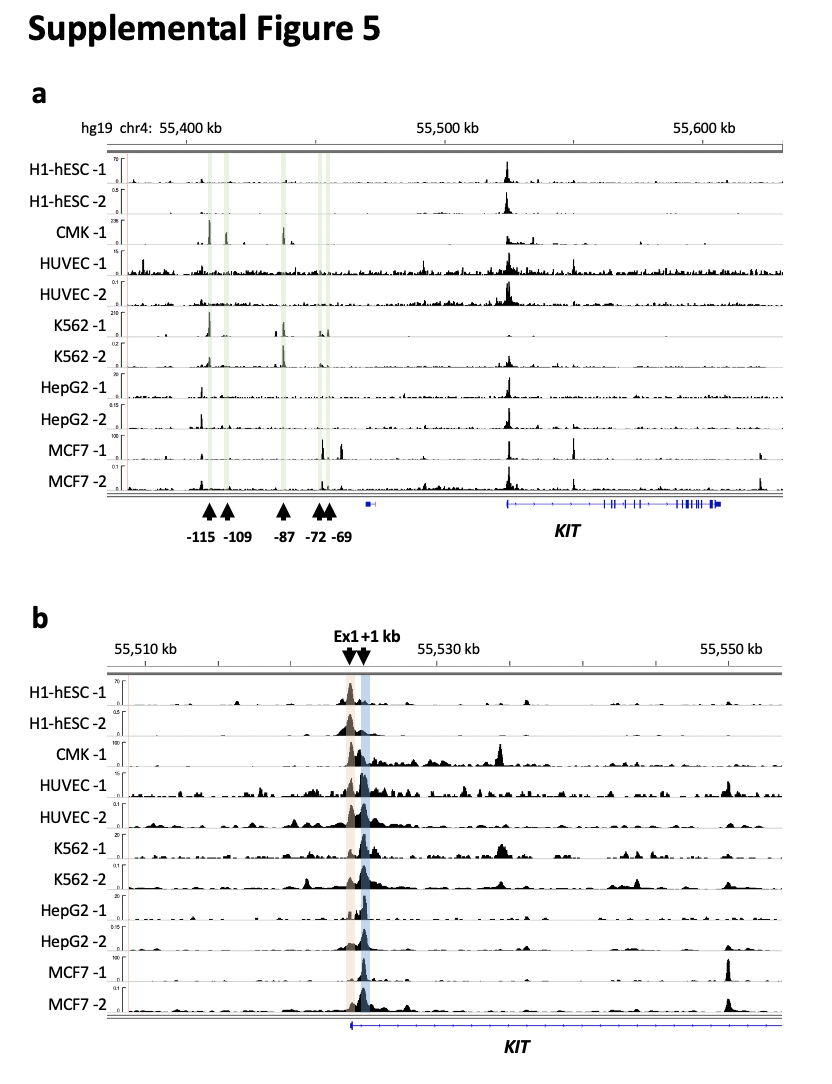
**

**Supplemental Figure 5. Open chromatin profiles of the *KIT* locus in various cell lines. (**a) Overall view of DNase-seq analysis at the *KIT* locus. Peak regions upstream of *KIT* detected in CMK or K562 are highlighted in green. (b) Zoomed-in view of the area around the transcription start point of *KIT* in Supplemental Fig. S5a. Exon 1 region is highlighted in orange and +1 kb region is in blue. These DNase-seq data were obtained from ENCODE repository at UCSC. The files used above are listed in Supplemental Table S3.

**
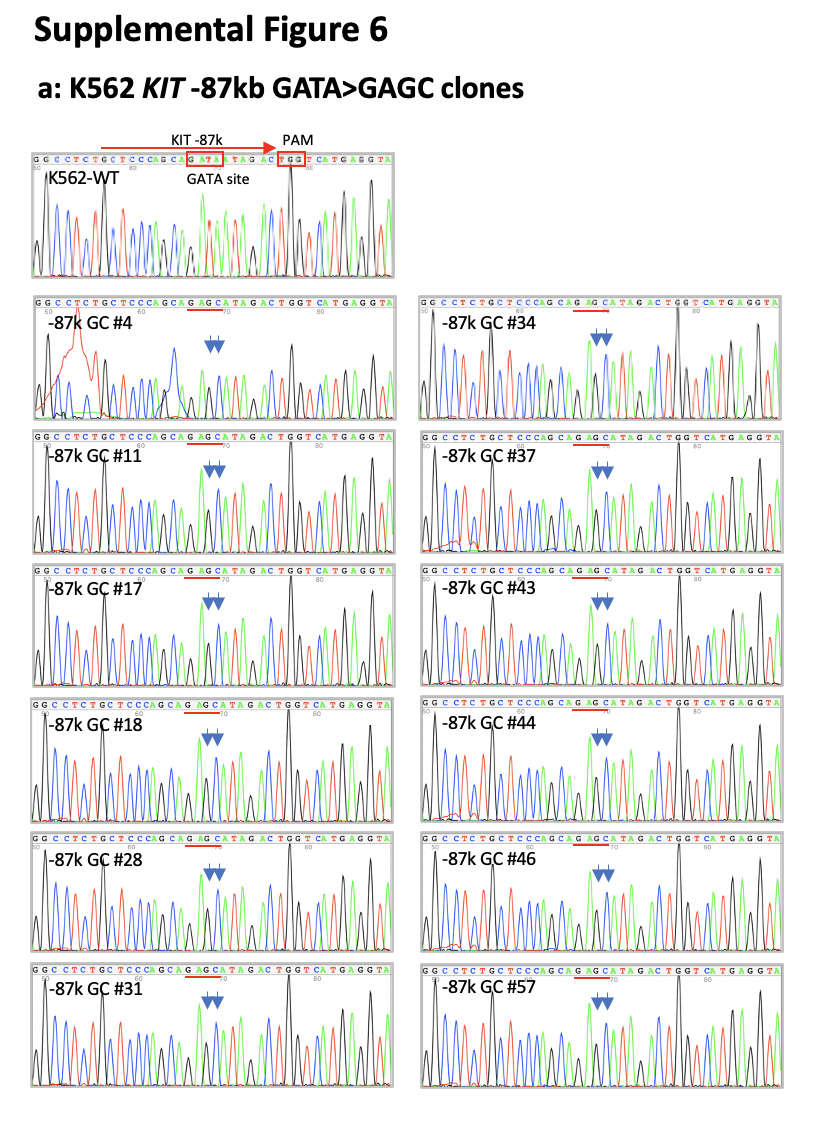
**

**
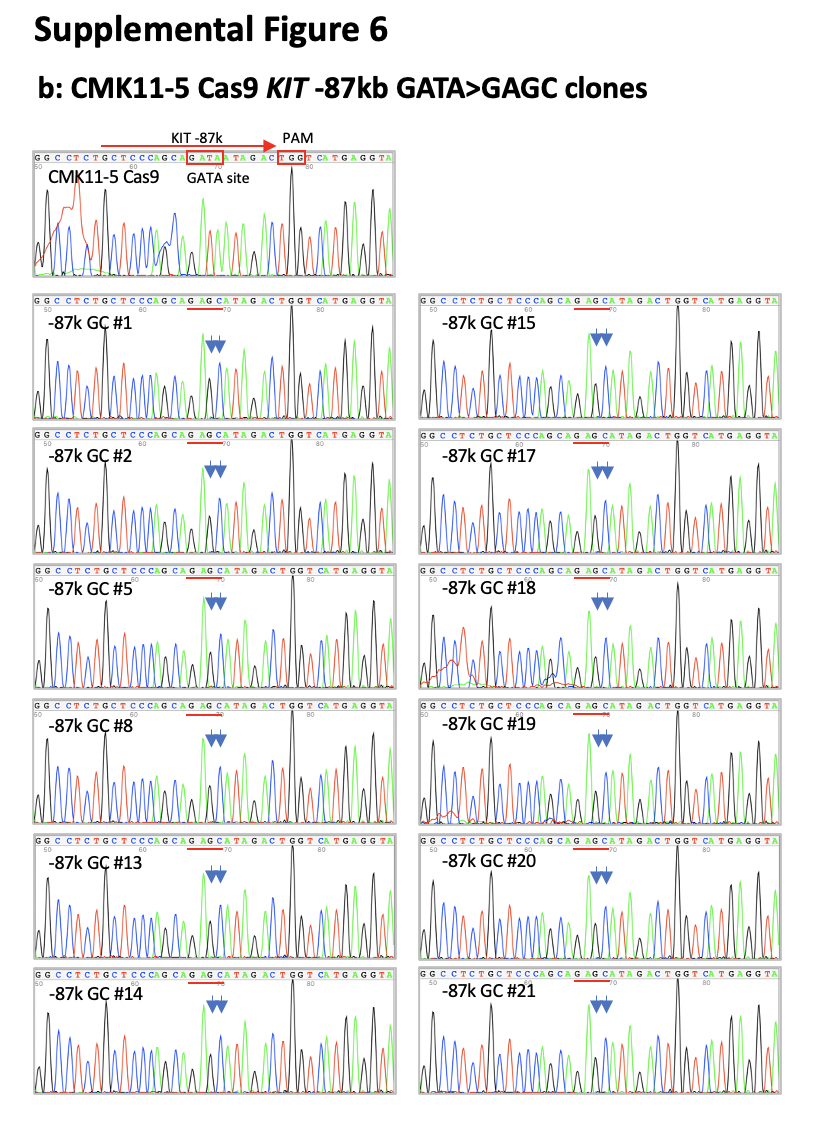
**

**Supplemental Figure 6. Confirmation of sequences in clones with genome-edited GATA sites in the *KIT* -87 kb region.** (a) Sequencing of twelve K562 *KIT* -87kb GATA>GAGC clones. (b) Sequencing of twelve CMK11-5 Cas9 *KIT* -87kb GATA>GAGC clones. The blue arrows indicate the positions of the base substitutions introduced via homology-directed repair (HDR). The red arrows indicate the target sequence for this genome editing. PAM: proto-spacer adjacent motif.


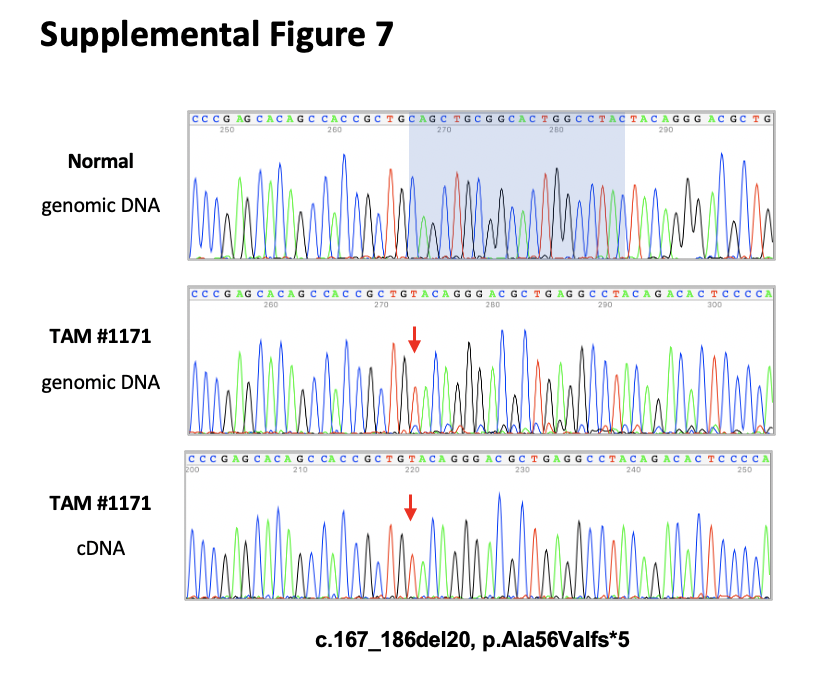


**Supplemental Figure 7. Identification of a GATA1 mutation in the TAM sample (#1171) used in this Sonication-based 3C experiment.** (Top) *GATA1* Genomic DNA sequence without mutation. (Middle) *GATA1* Genomic DNA sequence of TAM #1171. (Bottom) *GATA1* cDNA sequence of TAM #1171. The red arrows indicate the position of the mutation. The deletion range of the TAM sample is colored in blue on the normal genomic DNA sequence.

**
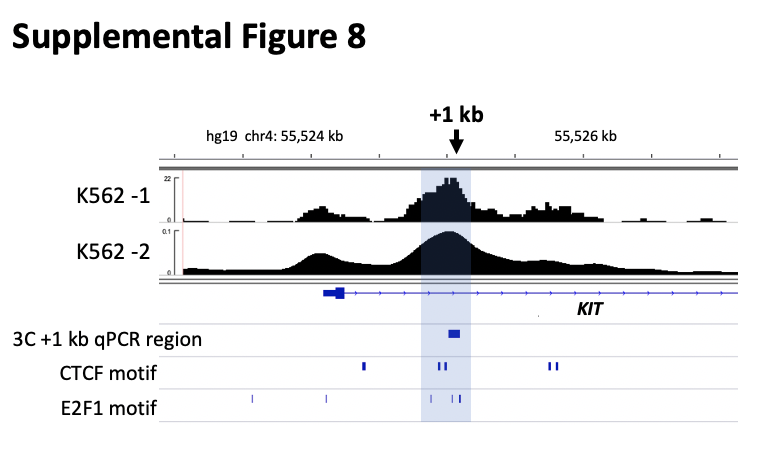
**

**Supplemental Figure 8. Location of CTCF and E2F1 binding motifs around the *KIT* +1kb region.** The top two columns show DNase-seq data in BigWig format for K562, the same as used in Supplementary Fig. S5. The column below the *KIT* gene shows the qPCR amplified region of the +1 kb region in the 3C experiment of this study. Motif scans were performed with JASPAR 2022 ([https://jaspar.genereg.net](https://jaspar.genereg.net/)) at 80% threshold. The bottom two columns show the location of the CTCF (MA0139.1) and E2F1 (MA0024.1) binding motifs, respectively. +1 kb region is highlighted in blue.


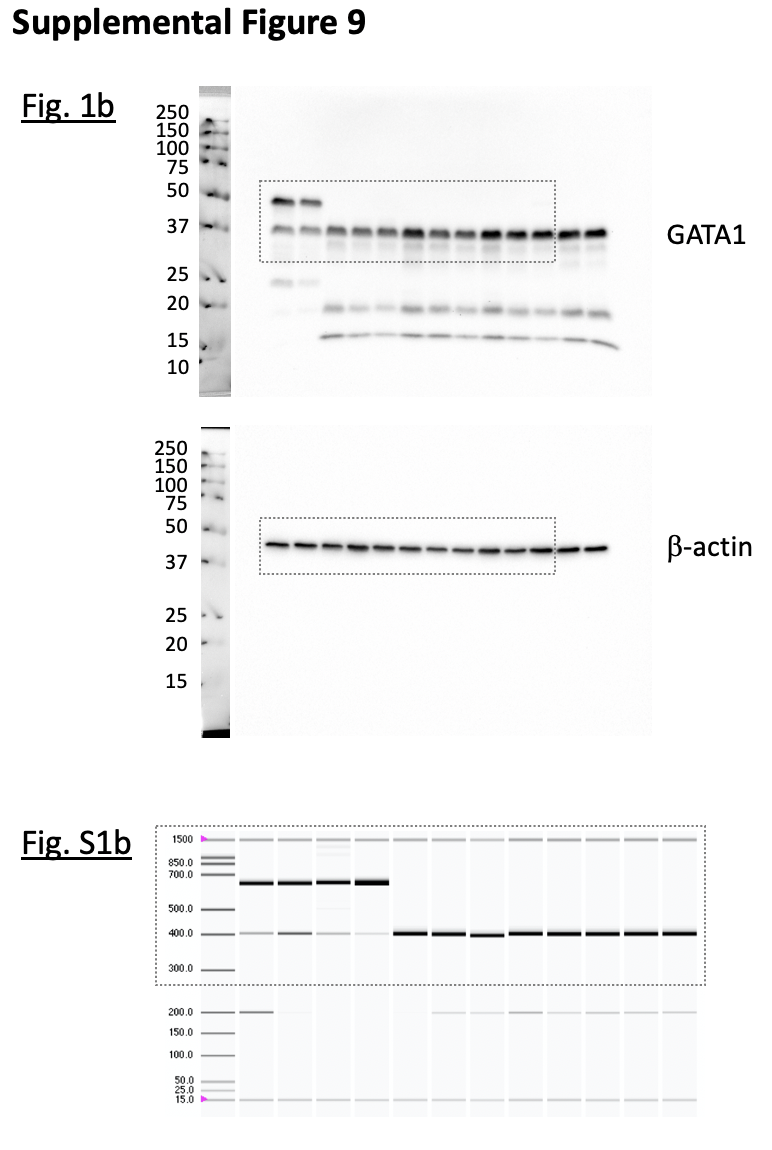


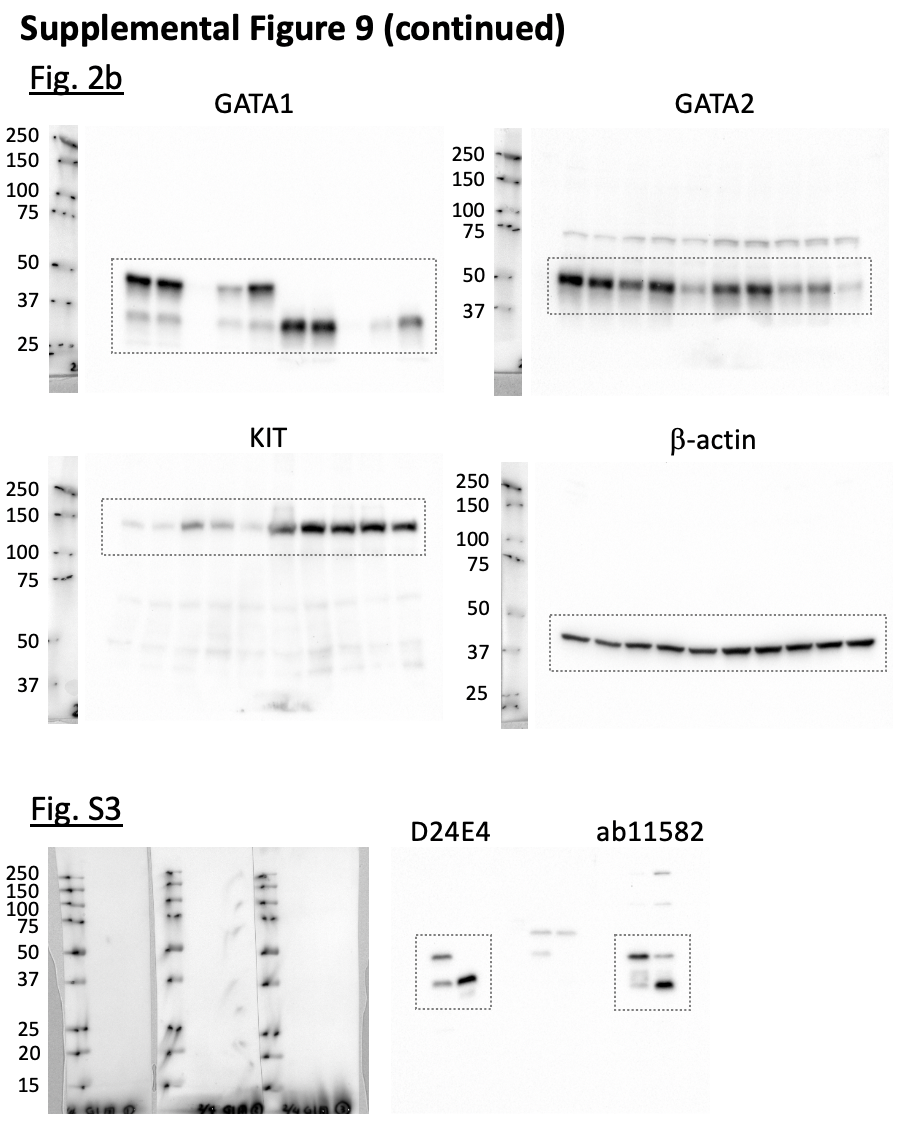


**Supplemental Figure 9. Uncropped images corresponding to Fig. 1b, Supplemental Fig. S1b, Fig. 2b and Supplemental Fig. S3.** The areas used for the cropped images are indicated by dotted squares.

**Supplemental Methods**

**CMK11-5 Cas9 establishment**

CRISPR genome editing is inefficient in CMK11-5 cells (data no shown). In order to improve the efficiency, we employed the Sleeping Beauty transposon system^1^ to establish CMK11-5 Cas9 cells, in which Cas9 is constantly expressed. We were provided the following vectors from Addgene: pCMV(CAT)T7-SB100 (#34879), pSBbi-GP (#60511), and pX330-U6-Chimeric_BB-CBh-hSpCas9 (#42230). The 4.3kb fragment of NcoI-EcoRI (blunt-ended) was cut out from the pX330-U6-Chimeric_BB-CBh-hSpCas9 vector. This contained the 3xFLAG Cas9 ORF region. This fragment was ligated into the NcoI-HindIII (blunt-ended) of pSBbi-GP, resulting in the pSBbi-GP Cas9 vector. To obtain CMK11-5 Cas9 cells, 1 μg of pCMV(CAT)T7-SB100 and 19 μg of pSBbi-GP Cas9 plasmid DNA were transfected to 2x10^6^ cells of CMK11-5 cells by 4D-nucleofector (Solution SF, program EH-100) (Lonza). After 24 h of transfection, selection was started with 400 ng/ml of Puromycin.

**Data acquisition from the ENCODE project**

DNase-seq data in bigWig format for CMK, K562 and representative cell lines H1-hESC, HUVEC, HepG2 and MCF7 were obtained from the ENCODE repository at UCSC (https://genome.ucsc.edu/ENCODE/downloads.html).^2^ These downloaded data were visualized with igv v2.8.0 software (Broad Institute).^3^ More details of the data sets used can be found in Supplemental Table S3. GTF-formatted expression data for the above cell lines other than CMK, whose expression data was not found, was also obtained from the ENCODE repository (Supplemental Table S4).

**References**

1. Kowarz E, Loscher D, Marschalek R. Optimized Sleeping Beauty transposons rapidly generate stable transgenic cell lines. *Biotech J*. **10**, 647-653 (2015).
2. Consortium EP. An integrated encyclopedia of DNA elements in the human genome. *Nature.* **489**, 57-74 (2012).
3. Thorvaldsdottir H, Robinson JT, Mesirov JP. Integrative Genomics Viewer (IGV): high-performance genomics data visualization and exploration. *Brief in Bioinform*. **14**, 178-192 (2013).
